# The scientist, the politician, the artist and the citizen: how water united them

**DOI:** 10.1186/s12302-018-0141-5

**Published:** 2018-04-16

**Authors:** Bernd Manfred Gawlik, Natalia Głowacka, David L. Feldman, Richard Elelman

**Affiliations:** 1 Water and Marine Resources Unit, Directorate Sustainable Resources, European Commission, Joint Research Centre, Via Enrico Fermi 2749, 21027 Ispra, Va Italy; 2 Department of Regional Bioenergy, Faculty of European Studies and Regional Development, Slovak University of Agriculture in Nitra, Tr. A. Hlinku 2, 94976 Nitra, Slovakia; 3 Environmental Institute, Okruzna 784/42, 97241 Koš, Slovakia; 4 School of Social Ecology, Department of Urban Planning and Public Policy, University of California, Irvine, 226F Social Ecology I, Irvine, CA 92697-7075 USA; 5 Fundació CTM Centre Tecnològic, Plaça de la Ciènca 2, 08243 Manresa, BCN Spain

**Keywords:** Urban water management, Sci-Art, Participatory approach, Social ecology, Water governance, Innovation uptake

## Abstract

The Urban Water Atlas for Europe constitutes an original overview of Urban Water Management in Europe, explaining and illustrating water in an unprecedented way and reflecting how water, the essence of life, flows through the arteries of our cities. Leading experts in water sciences and technologies, together with climate change researchers, have joined artists and children in order to show how thirsty our cities really are and how we can cope with their growing demand for the most precious resource of our planet. The result is the first major publication of the Science and Knowledge Service of the European Commission, the JRC, which within a movement stemming from its Sci-Art Programme seeks to explore the important opportunities arising from the cross-fertilisation between science and art. The Atlas itself establishes the benchmark for over 40 cities, both European and from farther afield, in 30 different countries, in a manner which permits a vast range of municipalities to confront one of the greatest global challenges by employing local solutions in order to ensure a supply of water for all. It contains 95 scientific indicators and parameters, over 700 graphs, original illustrations and never seen before photographs and combines the work of 40 contributors from 22 organisations. Yet, the true value of this publication lies in the process of ensuring that the underlying scientific knowledge is available for societal uptake. The resolving of conflicts which stem from an exclusive self-understanding of traditional natural sciences, the difficulty to communicate the purpose of technological solutions and the challenge to engage in peer-to-peer discussion between the sciences, politics and the citizen constitute worthy lessons for both environmental experts and their social science counterparts.

## Book details

Title: Urban Water Atlas for Europe. European Commission.
ISBN: 978-92-79-63051-4,
doi: 10.2788/114518,
Number of pages: 160,
Edited by: Gawlik BM, Easton P, Koop S,Van Leeuwen K, Elelman R, Glowacka N, Silva R,
Published by: Luxembourg: Publications Office of the European Union; 2017.

## Introduction

When it comes to water in particular, most people have a very profound and emotional connection with their place of birth and the regions in which they live. The ties often range from extremely personal memories and sentiments to a strong sense of belonging to the community in question and cultural identity [1]. However, when discussing science or, as is the case in this paper, urban water management, the emotional link and culture related to livelihood and well-being is all too often set aside [2, 3]. Emotions and science are not traditionally expected to interconnect and yet, we have in Europe, numerous examples where such human conditions and study areas have combined, in many cases because of water and have subsequently contributed to the prosperous development of cities [e.g. 4, 5].

Addressing the issue of the role of water in its local dimension, but with a global perspective regarding sustainability is key to the exploration of future opportunities and society’s capacity to overcome the obstacles facing cities who seek to implement effective urban policies which will guarantee sustainable management, effective governance and a stable supply of water in municipalities [6, 7]. A number of international environmental and water platforms address the topic of water with diverse initiatives, international project proposals, collaboration and improved transversal synergies such as the UN-Water, the Global Water Partnership, the Global Water Research Coalition or the International Water Association, to name but a few.

However, at a social level, the issue of water has still not established itself in the consciousness of the man in the street, despite it being the principal environmental and probably socio-political issue of the 21st Century. This paradox is recognised and considered by the Sustainable Development Goals formulated within the UN 2030 Agenda for Sustainable Development [8]. It is a situation, which demands that society learns to communicate the complexities of such issues in a clear, transparent manner which is comprehensible to all. This responsibility pertains to all relevant actors equally including scientists, politicians and municipal administrations.

Cities are the closest administrative entities to the citizen. Their proximity and capacity to interact with the uninitiated layman demands that they undertake the role and, indeed, accept the obligation to present water as the vital environmental issue that it is. This exceeds the mere establishment of specific Sustainable Development Goals such as SDG 6 (water) and SDG 11 (cities) [8]. If municipalities are capable of creating a profound social awareness as well as implementing true citizen engagement, a social consensus is then possible and as a result a cross-party, political unity. This would lead to the political continuity at municipal, regional, national and supranational levels, which is required to permit environmental policies to demonstrate their true worth [9]. Contrary to what is sustained in the Smart-City approach, sustainable urban living cannot become a reality by attending exclusively to issues of public transport, energy and ICT [10]. Water, waste and urban biodiversity, as expressed by the Urban Water Agenda 2030 [11], must be considered equally and additionally in the group of sectoral interests that will determine the future and quality of urban life.

Having understood that smart cities and sustainable urban areas and communities are key research and demonstration areas to be examined throughout the world, the European Commission’s Joint Research Centre, the Network for Water in European Regions and Cities (NETWERC H2O) and the partners of the H2020 Project: BLUESCITIES created the Urban Water Atlas for Europe [12] as an original tool with which to bridge the gap between scientific understanding and societal knowledge, with the ultimate goal to promote, support and improve the application of sustainable practices in the use of water at a regional and municipal level.

The Atlas, which was launched at the Ministerial Meeting of the Union for the Mediterranean in Malta in conjunction with the UfM Ministerial Water Declaration [13], constitutes a remarkable interdisciplinary effort. However, the purely editorial effort aside, it is worthwhile to examine the processes and dynamics behind the making of the Urban Water Atlas for Europe. The Atlas was the result of a unique experience that deserves dissemination to the scientific community represented by the readers of Environmental Sciences Europe and this *behind the scenes, making of* report is intended as an invitation to consider how knowledge of environmental sciences can be made accessible to a broader audience, thus overcoming the substantial gap between general perception and reality.

## The invisible urban water challenge

Despite being irreplaceable, water is only renewable if conscientiously managed. Society must be open to water management innovation, especially in cities and towns—the urban areas, which are home to an ever-increasing majority of the world’s population.

Municipalities (or cities as they tend to be generically and erroneously named) have historically had little or no say in the on-going international dialogue concerning water affairs despite being identified by the majority of the leading international organisations such as the United Nations, the Organisation for Economic Cooperation and Development and the European Union as key figures in the move towards an environmentally sustainable society [14–16].

Supranational entities bereft of the opportunity to engage to such an intimate degree with citizens openly recognise that local administrations are the most important interlocutors with the general public and are, thus, essential actors in the creation of public awareness, social consensus and the political continuity required so as to be capable of ensuring that long-term water policies may achieve their full potential [11, 17].

Municipalities are centres of economic growth, employment, creativity, culture and innovation, but they are simultaneously producers, consumers and sources of a host of global environmental problems. The initiatives to safeguard water resources often omit to underline the importance of municipalities, whilst the challenges and solutions regarding sustainable water use, energy and resource recovery will predominantly reside in cities [11, 18]. Therefore, one can state that there is a real and urgent need to ensure that the adequate conditions in order to ensure that our municipalities become the catalyst for improved urban management of our water resources exist. Municipalities could and should provide local solutions to global issues if and when they develop a coherent, long-term integrated strategy and implementation plan that encompasses transport, energy, ICT, solid waste, climate adaptation (heat islands, urban flooding, and water scarcity), water supply, wastewater treatment, air pollution, and urban design.

While urban residents crave green and blue space, to date, many cities have failed to provide adequate remedies to their citizens, or to prioritise this issue on urban agendas [5].

The causes of the socio-political invisibility of urban water issues cannot be explained from one perspective alone. What is clear, however, is that an unprecedented effort on the part of all engaged stakeholders to abandon traditional approaches in favour of more courageous and open strategies is a necessity.

### The conflict between water sciences and urban politics

Not unlike other environmental issues for which ideological preconceptions sometimes pre-empt scientific fact—such as climate change or even renewable energy [19]—water science and politics is sometimes characterised by deep distrust and a lack of consensus, e.g. as illustrated by the Flint Water Crisis [20, 21], the Salton Sea Project [22] or the on-going debate in Europe regarding the minimum criteria for water reuse. This has also contributed considerably to the erosion of trust between citizens and environmental policy makers at an EU level. Trust requires dialogue and dialogue can only advance with trust [23]. The topic of water comes to the attention of the public in a negative form, indeed as a threat: drinking water (despite the abundance, high quality and the significant and objectively measurable improvements achieved through the European Water Framework) is perceived as being contaminated and as an object of speculation, manipulated by the financial sector [24]. Meanwhile, the scientific community often barely conceals its contempt for politics or political processes which are viewed by the former as a class of “*evil force*” or a form of hostile species who act in a manner that is completely detached from the society they are supposed to represent (see e.g. [25, 26]).

This reaction is symptomatic of a more profound and serious situation, the cause of which must be identified and understood if it is to be overcome. In 2015, Green published a rather unusual scientific paper, which investigated this difficult relationship between science (in this case marine science) and political processes, employing the example of the definition of fishery quotas [27]. Green correctly stated that “*the role of fisheries science is not to promote the ‘side’ of fish, but to ensure that fishermen are able to catch fish sustainably”.* In this remarkable analysis, Green identified the mismatch between the political need for scientific certainty versus the notion of probability, which dominates the world of scientific thinking as the principal cause of this conflict. The aforementioned confrontation is further exasperated by the very manner in which science produces knowledge, i.e. a closed approach by which recognition is sought exclusively from peers and not from the public nor their elected political representatives.

Therefore, one can state with some confidence that with regard especially to environmental and sustainability issues, science cannot be left solely to the scientists and that society as a whole must be engaged. Confusion is all too often generated by *populist science*, such as that observed in the mass media, expressed by environmentalists who frequently present themselves as an authority on the issue in question and obtain the recognition from high-level political actors that would not be forthcoming from their scientific peers [25]. Meanwhile, more orthodox researchers prove to be overly concerned with the relative values of the measurement of uncertainty rather than with translating their knowledge into understandable advice [28].

The result in relation to urban water management is that science is failing to communicate clearly with regard to the appropriate priorities for the local policy agenda. Thus, one finds oneself confronted with grave anomalies. For example, the occurrence of compounds of emerging concern in tap water (e.g. [29]) or connected water resources [30] has received a lot of attention whilst the fact that most European cities loose considerable amounts of pure drinking water due to leakage and antiquated infrastructures hardly raises an eyebrow—on average 25% exceeding even 40% in some cases [31]. Despite the fact that renewable water is abundant in Europe, signals from long-term climate and hydrological assessments, including population dynamics, indicate that there was a 24% decrease in renewable water resources per capita across Europe between 1960 and 2010, particularly in southern Europe [32]. Droughts and their effect on vegetation are still perceived as a specific problem for the Mediterranean region whilst the systematic reuse of treated wastewater or rainwater in an urban setting is regarded as an outlandish practice in a number of non-European locations [33]. The perceived abundance of freshwater resources means that such strategies are considered as being politically dangerous and thus to be avoided.

Nevertheless, positive examples of local, broad sectorial engagement do exist (Table 1) and interestingly, they are not confined to traditionally trend-setting municipalities such as Amsterdam, Copenhagen or Berlin.

**Table 1:** Lesser-known examples of urban water management practices establishing a link between science and technology and the citizen

City	Country	Example
Malmø	Sweden	Nature-based solutions as a political and social construction or collection of actions rather than simply a technical problem [34]
Slupsk	Poland	Water democracy and Pomeranian Landscape Park Complex in Slupsk [35]
Lodz	Poland	Innovative Urban Water Governance to reconcile urban growth and integrated urban water management [36]
Milan	Italy	Wastewater Treatment Plant Nosedo used for local arts exhibition “arte da mangiare—mangiare arte” [37]
El Port de la Selva	Spain	Using water recycling for aquifer recharge for indirect potable reuse in coastal areas [38]

### The distance between water science and the citizen

Traditional *hard* sciences have been of paramount importance in raising public awareness of sustainability as an issue, but the same sciences fail to provide adequate solutions—in other words, 20th Century solutions will not help society to face the challenges of the 21st Century. Unlike any other environmental domain, water is a topic in which it is proving especially complicated to create a link between climate change processes and the necessary adaptation measures needed. It would appear that people believe that the planet must be saved whilst ignoring the fact that this also entails saving the human race. Why is this so? Why are simple notions such as the fact that people cannot survive without water or that one does not build houses near to a water course not leading to a broad social consensus that action at a local level is imperative? The answer is frustratingly simple. Such basic premises simply do not reach large sectors of society and one of the key reasons for this is that scientists often do not possess the necessary democratic awareness that their knowledge is influenced by and influential to policy [25]. Democratic awareness does not mean that scientific truth should be subject to public approval. It means that the power derived from knowledge is not accessible to the vast majority of citizens. Thus, the extraction of science from the grip of academia appears as urgent a task now as extracting knowledge from the religious establishment was in the 15th and 16th centuries.

The risk for decision-making is that in a *post*-*factual world,* if scientific truth is not reaching society, society creates its own truth based on perception and interestingly, emotions [39]. To understand this link, one must bear in mind that sustainability issues such as water become more socially relevant the closer they appear to human habitats, i.e. the urban settlement [40]. Studies have deduced that to address such challenges with the required sensibility and urgency, modern (natural) science must construct bridges to social problems that arise from income and power inequality [41–44].

### The urban water dilemma

The Urban Water Agenda 2030 [11] acknowledges that sustainable urban water management is fundamental to ensuring the quality of water for human use and preventing pollution of water in cities. This includes reducing water abstraction to a sustainable level and achieving good ecological status of water bodies, ensuring efficiency of the urban water system, sustainability of urban water infrastructures, flood prevention and raising citizens’ awareness of water as an essential, precious resource.

Yet, at a local level, water management is generally relegated to that of a simple matter of infrastructure, combined with other municipal facets, many of which prove to be of greater political interest, especially within the EU. Water services and water sanitation are taken for granted and only succeed in attracting attention when problems or service failure appear. It is what can be described as the *catastrophe effect*. The 2017 droughts that hit the Mediterranean area provide a typical example whereby a water supply shortage in the city of Rome was, for a few short weeks, the reason why water became an important news item, only to return to oblivion once normal service was resumed. The reasons and causes for the aforementioned shortage have their origin way back in time. The reactive prevails over the proactive. Water is not a political priority until it ceases to be readily available. This is the urban water dilemma. Proper management requires a long-term, strategic approach that reaches far beyond any mandate of a city administration and this approach needs to be based on an undisputed consensus regarding the municipality’s water priorities.

The role of science is to assist the city in defining the priorities and presenting them to society, but, in general, this does not happen. The private water sector alone is not in a position to undertake the type of assessment required. Indeed, all urban water assessment tools (Table 2) ultimately are orientated towards (a) commercial interest or (b) the establishment of comparative municipal rankings instead of seeking to assist urban administrations in recognising and tackling the underlying urban water challenges.

**Table 2:** Selection of urban water assessment tools

Name of indicator	Description
City Blueprint	Aims at overall sustainability of Integrated Water Resources Management (IWRM) in municipalities and regions. A main focus is on the integration of water, waste and climate adaptation in cities. Uses mostly data from International organisations [45–47]
Urban Water Blueprint	Defines the watershed conservation potential for 534 cities evaluating five conservation activities: reforestation, agricultural best management practices; Riparian restoration, Forest protection, Forest fuel reduction [48]
Water Sensitive Cities Index	The WSCI is designed to benchmark and rank cities based on water sensitivity performance. It also set targets and inform management responses to improve water-sensitive practices. It is supported by a web platform to enable visualisations of benchmarking results for a range of audiences, including policy makers and service providers [49]
Sustainability index for Urban Water Management System	The sustainability index can be seen as a precursor to the aforementioned City Blue Print. It addresses 74 criteria of urban water management: social, economic, environment and engineering [50]
Sustainable Cities Water Index	The index focuses solely on water through three main factors, each with their own sub-indices: resiliency, efficiency and quality. Each city has been ranked based on these three tenants in order to raise awareness of the role of water management in defining a city. The tools are intended to guide future improvements, investment and water sustainability [51]
Urban Water Utility Sustainability Framework	Initial results of U.S. urban water utility sustainability indicate that many of the preliminary indicators are closely connected to energy. Further study will finalize the set of recommended indicators, but it is clear that energy will play a key role in the “snapshot” assessment of urban water utility sustainability [52]
Water Use and Climate Index (WUCI)	The WUCI analyses and presents data of 142 cities and the re-grouping of them using a hierarchical cluster analyses [53]
Sustainable Development of Energy, Water, and Environment Systems (SDEWES) Index	This tool addresses the sustainable development of energy, water and environment systems through an integrated approach with the goal to foster policy learning, action and cooperation for sustainable development in cities across the globe. The index rates cities based on 7 dimensions, 35 indicators and 20 sub-indicators. It is currently used for 58 cities [54, 55]
Swedish Sustainability Index for Municipal Water and Wastewater Services	Developed by the Swedish Water and Wastewater Association (SWWA), this index is a tool for analysis and decision-making over the short and long term. It aims to prioritise actions and investments, monitor improvements, create a basis for strategic planning, and to analyse the needs of municipalities. It does not rank municipalities but aims to provide the municipalities with their own results to build a strong and context-based solution to water and wastewater management [no citable reference]

The key to overcoming such obstacles lies in the creation of synergies. If the urban water issue is not only understood as an environmental challenge but also as a social threat, the appropriate solutions must also address the social issues in question, thus increasing the impact of envisaged measures as well as enhancing the societal acceptance and endorsement of the proposed (technical) solution.

## The opportunity: understanding the role of art and culture

Science and technology are considered in Europe (and beyond) as principal sources of nation-state and religious competitiveness and sustainable potential [56]. To optimally exploit this potential is in the efficacy of transforming advances of science and technology into pecuniary benefits through entrepreneurship-enabled innovation. This, however, depends crucially on the speed with which innovation is adopted by the society in question. In other words, it is essential to connect ideas and solutions with markets and investors. This link between basic and applied research with the market, employing technology transfer and commercial mechanisms has become the subject of increasing attention, the launch of the digital economy being the latest of a long list of such developments. Yet, when it comes to sustainability-related issues and in particular with regard to water, this evident interaction encounters difficulties in the form of cultural, psychological or social resistance [1]. Thus, for instance, despite the evident advantages of a direct potable reuse of treated wastewater in water scarce regions, social acceptance is proving elusive to say the least due to a perception of risk [33].

Although comparatively few in number, there do exist positive examples of potable reuse practices and all of them can demonstrate true and honest citizen involvement in the respective water reuse projects [57]. Analysing these examples, one understands the need to go beyond a pure science-based approach and to admit that not only science, but also art and culture are reason-based and need to be used to reconnect the environmental function of water with its cultural dimension [1]. From this, one can derive the necessity for a more integrated approach to *knowledge*, an advanced knowledge system, which includes science, arts and broad social culture.

The original objective in the creation of the Urban Water Atlas for Europe was to describe a collection of urban water management practices and to scientifically analyse a selection of cities with regard to the identified urban water management practices and needs. The goal was to disseminate lessons from these experiences to municipalities elsewhere. While primarily dependent on science for its investigation and reliant on political diplomacy for connecting cities—the endeavour also represented an important foray into another form of interaction, the relationship between science, politics and art. The use and dissemination of youth-generated art became in this endeavour a means of uniting diverse municipal authorities from very different regions and countries in their mutual ambition to establish sound water management at a global scale. It proved to be a venture that demonstrated its capacity to help address the fundamental aspirations of people everywhere for clean water. It also demonstrated that it could lead to the creation of a future group of socio-political leaders armed with the necessary sensitivity so as to be able to achieve such aspirations.

### The Dubrovnik process

As a stepping-stone towards a more integrated and collaborative approach involving medium-sized municipalities interested in improving water resilience, a multi-stakeholder workshop was organised in Dubrovnik (Sept 2015) facilitating the encounter between mayors or other representatives of municipal administrations. The focus was placed on Eastern Europe, the Danube Basin and the Near East. The participants combined their respective forces and know-how in order to investigate the importance of the role of local administrations in resolving common urban environmental issues, to ensure improved synergies between cities and to employ tools for integration and implementation, stakeholder engagement and international networking. The substance of the Dubrovnik process was concern over inequality, diversity and engagement.

Inequality is an environmental issue just as environmental degradation is a social issue (forming a *socio*-*ecological nexus*), and solutions must address them jointly through principals and institutions rooted in justice [40].

Communities with the greatest and most diverse citizen participation are often resilient and strong. Engaging citizens to address common issues is essential for educated decision-making. No other field of urban infrastructure intervention benefits as much as water infrastructure and to a lesser extent waste management from active citizen participation and engagement [9]. In this context, the successful deployment at a municipal level of innovative and novel solutions, be they technology-based or concerned with governance, is of vital importance to establish the EU as a world leader in such technologies and in its attempts to create green jobs, healthier societies and improved economies.

Public engagement is key to ensuring the necessary political continuity beyond a single mandate in a local administration. To achieve this, it is also vital that a collective knowledge concerning the solutions to be selected and implemented is cultivated and at the basis of any consensus-building process [1, 9, 44].

At Dubrovnik, it was agreed that the exchange of best-practices in urban water management can most effectively be achieved by establishing a direct contact between cities of similar profiles and needs. This mechanism was successfully employed in an approach dubbed *Winning*-*by*-*Twinning* [12].

This common desire was channelled into and expressed through the Dubrovnik declaration of Intent, presented at the end of the workshop and subsequently signed by representatives of the participating cities together with the process’s social and scientific contributors [12].

The Dubrovnik declaration and the Dubrovnik process captured the attention of many supranational and national entities, and a number of important cities and regions have signed, or are in the process of adhering to it. The workshop participants and signatories of the Dubrovnik declaration declare their intention to collaborate with other cities, to involve the local administrations and stakeholders in a participatory context and to formulate the regulatory framework that will promote the successful design and implementation of novel and smart urban water management solutions leading to more resilient cities. In collaboration with experienced experts and academics from a wide range of disciplines, the participating municipalities are committed to designing and monitoring climate change stress and sustainable water resource management.

During the workshop, the participants were shown how to employ tools for integration and implementation, stakeholder engagement and international networking whilst emphasising the importance of multi-level public administration and multi-sectoral dialogue. The key aim is to support municipal integration and inter-municipal cooperation.

### The science and art effect

The Dubrovnik process also led to a series of initiatives involving schools, thus creating an elegant way to engage not only with children but also their families and ultimately local society in general. The writing of the Urban Water Atlas witnessed the evolution of this approach into a tool to stimulate inter-city collaboration through what is commonly known as the Science-and-Art Movement (SciArt). The idea of SciArt is actually very simple yet ground breaking. Assuming that the creation of art as well as the discovery of knowledge in research involves the same process of human reasoning, SciArt tries to stimulate a cross-fertilisation between the two domains with the objective of remodelling perspectives and paradigms regarding the traditional manner of working in, and using science. Successful examples of such a transformative move are, for instance, the ARTS-AT-CERN Programme or the Science Gallery pioneered by Trinity College, Dublin.

To unlock this potential for the creation of the ATLAS, the emerging Polish artist Natalia Głowacka, who simultaneously works as a biotechnologist, created seven paintings known as *The Water Cycle*. This is a series of paintings which illustrate diverse dimensions of water: the political challenge, equality, the water-energy nexus, the connecting aspect of water, its force as an element of nature, its attributes within a divine trinity of water, vapour and ice and hence its role as a common element to the spiritual reality of mankind. The paintings of Głowacka were used to inspire school pupils aged from 8 to 12 years for an international school competition on water. The competition was organised simultaneously in the cities of Amman, Jerusalem, London, Manresa, Sfantu Gheorghe and Istanbul and the children, motivated by the aforementioned art were encouraged to visually express their own views and feelings on water.

Efforts to directly link art, education, and water science are becoming increasingly popular. An example, directly sponsored by a city, is the New York City Department of Environmental Protection’s Annual Water Resources Art and Poetry Contest which is open to 2nd to 12th grade students attending either public, independent, charter or parochial schools or tutored at home. The participants are invited to create original art and compose poetry that reflects an appreciation for the region’s shared water resources, and student participants are annually honoured at a city-wide event. The contest is closely aligned with both New York State Learning Standards and New York City Performance Standards for elementary, middle and high school students in the so-called STEM (science, technology, engineering, and maths) fields, as well as the *Common Core State Standards* for English Language, Arts & Literacy in History/Social Studies, Science and Technical Subjects.

Efforts to understand the global water crisis through arts education are being pursued by many universities. Pennsylvania State University’s African Diaspora Water Crisis Curriculum Project, funded in part by a grant from Pennsylvania State’s Africana Research Centre, is but one of the number of notable instances. The project aims to develop and implement arts-based high school curriculum and instructional resources in response to the sufferings of some 345 million people in Africa and 32 million in Latin America and the Caribbean who lack adequate access to potable water. Curriculum and instructional resources are developed in collaboration with teachers in schools with predominantly African-American student populations for use in international programmes. Students from throughout the country visit Pennsylvania State and attend classes in art education, studio art, and art history, in addition to learning how to make ceramic water filters. A key aim of the project is “*to enable African American students, their classmates, and their teachers to situate themselves critically within the African Diaspora through direct exploration of the global water crisis through artistic, scholarly and socially engaged practices*”.

Art is capable of conveying complex principles related to water in a visually compelling way that connects imagination, emotion and reason (the latter, exemplified by science). At the same time, the arts—visual as well as performing—communicate these principles on a person-to-person, individual level. As a result, the role of the arts in disseminating powerful ideas and principles regarding water, to diverse groups of people, and across national boundaries, may in some cases be more effective than that of more conventional forms of political communication.

Neri Oxman tried to explain the Science-Art effect by comparing it to the Krebs Cycle in Biology and postulating an analogy to an effect of *creation of creative energy*, when science and art meet [58]. Indeed, following this reasoning one understands that the relationship between art and science is more complex than it appears at first sight and that for instance, engineering and design are related disciplines in the transition from pure science to art. Some aspects also reveal a hierarchy within this relationship:

- Art as propaganda for science: Certainly, the most commonly used (and misused) function where the *illustrative* function of art is used to *communicate science*. In this lowest form of interaction, art serves science.
- Science can be employed to provide innovative tools of artistic expression or simply to obtain access to funding: artists exploiting science funding schemes.
- A more interesting and novel aspect in this relationship from a societal perspective is the fact that art can act as an early warning system for science, as it addresses, describes and channels concerns, fears and other reactive social perceptions which regard scientific developments as a threat. Indeed, modern science fiction and dystopias may be considered as illustrations of this *precautionary* function.
- The noblest form of interaction is co-creation based on an equal exploratory partnership, where art catalyses new scientific knowledge and science is converted into original, ground-breaking artistic expression.

The experience of producing the Atlas featured all the above mentioned modes of interaction, and to the surprise of the authors, a further, unexpected aspect leading to what the authors have named *SciArt Water Diplomacy*—a peace-building dialogue between conflicting parties based on the topic of water, supported by both scientific and technological input, but catalysed within a cultural and artistic context. In other words, the dialogue was the Dubrovnik process in its practical application. To understand this effect, one has to look first at the issue of water from the perspective of both a scientist and an artist (Table 3).

**Table 3:** Water: the scientific perspective and the artistic perspective

Scientific perspective	Artistic perspective
Human life begins in an aqueous environment. Water provides a safe environment for the developing organism, ensures the freedom of movement, protects against too much stimulation from the outside world. Water is involved in all systems of all living creatures. Our blood and our tears, all come from water. From a chemical point of view, we can consider the issue of water from many perspectives. Among all molecules, water is one of the most unique, which is perfectly known to man. It is one of the most important molecules to our biological systems. Water is a crucial component of the human body, it regulates body temperature, and, keeps our skin soft and our organs hydrated. Water is the principal constituent of a body’s cells. However, there are still many questions to be answered, about the importance of this most significant element of the body and one’s everyday diets	Water. Majestic beauty. The topic of water is deeply inspirational. The process of capturing and painting water is quite complicated. One of the most beautiful and magical elements in the art of water is light, which passes through and creates the reflection of water. From the artistic point of view, capturing water is not easy at all, due to its irregular structure and unpredictable behaviour. To achieve the desired effect, an artist needs to deal with sparkles, reflections and light to capture and paint the colour, flow and rhythm of water. Forms and shapes which can be created by water are countless. Water is mysterious, wild and uncontrolled. At first, water seems to be transparent, but this is simply a superficial first impression. In fact, we can identify a broad colour scale, in the movement of the surface of water. The experience of water develops new artistic expressions. Returning to a human’s initial experiences of being in water, feeling the softness and warmth of water, brings a sense of security, which one instinctively feels reflects the experience of a mother’s womb. This feeling touches all human beings. It is impossible for one to not recall from where one has originated

**Fig. 1:**
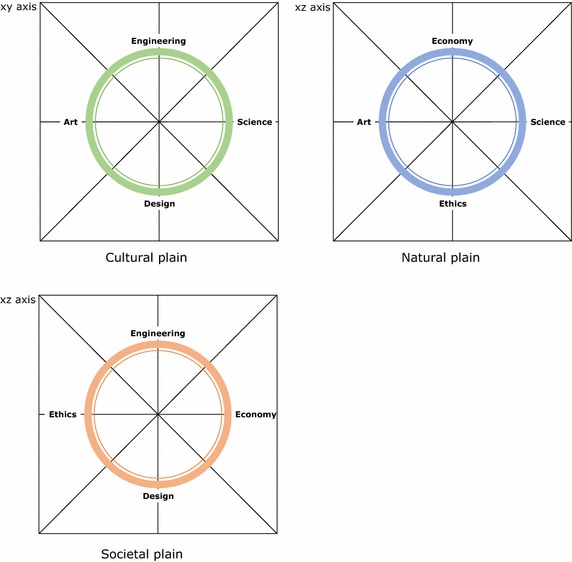
Creativity as a 3D model between art and science

It should be noted that Oxman’s Krebs Circle of Creativity contains a significant flaw in reasoning, because it actually omits that art and science, despite being both connected by reason, are two fundamental opposites or pathways. In more simple terms, the circle is rather to be seen as a sphere, where three planes collide (Fig. 1).

**Fig. 2:**
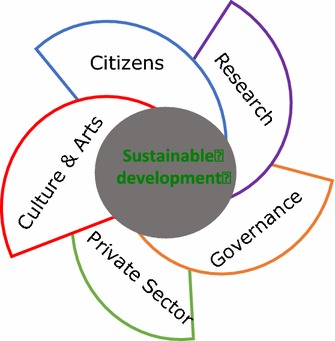
Cross section through the quintuple helix approach for sustainable development and innovation uptake

Applying this concept with regard to the social uptake of innovation related to sustainable development—and this is what was contemplated in the ATLAS—one obtains the *Quintuple Helix Approach* [40]. The traditional *Triple and Quadruple Helix* structure between research (Science), the private sector (Economy) and Governance, cannot be conveyed to the citizen without art and culture (Fig. 2). Social acceptance of sustainability-orientated innovation, despite a common agreement regarding its propriety, does not take place and the behaviour of the citizen does not alter. Only if and when the *Quintuple Helix* mechanism functions is an understanding between stakeholders created and a mutual trust constructed. These are the key conditions for any peace-building process.

## Conveying the message—the ATLAS

The Urban Water Atlas for Europe provides a visually compelling overview on how water is managed in European Cities. It explains and illustrates in an unprecedented way, employing the aforementioned *Quintuple Helix* Approach, how water flows through the arteries of contemporary cities. The remarkable feature of the ATLAS is that together with its highly interdisciplinary character that encompasses fourteen professional perspectives, it succeeds by the inclusion of art and a strong regard for citizen-engagement to make the topic of urban water management not only interesting but also emotional and hence more compelling to the layman, stimulating an interest in the reader to discover and further investigate the issues described.

## Outlook and perspectives

Europe has a long tradition of inter-municipal cooperation, which in recent years has become even more essential to the public due to growing aspirations or accountability and representativeness. Urban areas, which have been in the vanguard of the development and implementation of environmental policies, have a vital role to play as leading examples to more sceptical or less experienced counterparts. A single municipality benefits from the support and mutual collaboration of others, with no single community having all the answers to its problems. The combined learning of different communities can guarantee access to tried and tested methods to achieve sustainable urban water cycle services.

While national and international institutions are effective at identifying the broad global trends to be addressed, particularly in addressing access and management of such a vital resource such as water, local administrations are principally responsible for the implementation of policies, whilst engaging citizens. The municipality is the level at which administrations can interact most effectively with citizens and ensure they are informed. An informed citizen has greater interest and is better able to express any concerns. If their concerns are not alleviated, this may provoke fear. Knowledge and in particular knowledge about a more sustainable management of water must therefore be accessible in order to ensure that citizens together are capable of creating an informed social and political consensus (as opposed to a popular consensus based on ignorance), without which even the most ambitious political, economic, and social measures are doomed to failure. Many of the aspects of a successful urban transition require time and money. Public opinion, a culmination of individual expressions of preference, becomes votes and votes are converted into executive power, which is accountable to the general public. Active civic participation at the municipal level in alliance with technical expertise, becomes the foundation stone of a new, environmentally sustainable community.

Access to clean water is one of the principal issues the world faces. Humans prospered for thousands of years without electricity, motorised transport and many other examples of what we now consider to be basic ingredients of our existence. But without water, there is no life. Europe in general enjoys an ample supply of water, but forms part of a global reality which if not addressed now may lead to the most catastrophic of scenarios in a not-so-distant future. Climate change, land use modifications and demographic evolution have already demonstrated their devastating results. Whilst Europe has witnessed damaging floods and water scarcity in some regions, other regions in the world have faced severe drought and famine.

Europe has an important role in the mitigation of the threats to our planet. In the same way, every city and municipality must recognise its own contribution to the problems whilst assuming a responsibility to promote a more sustainable global environment. The way forward is through successful urban governance combined with stakeholder awareness and participation, and systematic collaboration between cities of all regions. The evidence cannot be ignored any longer but the future need not be bleak.

As shown in this paper, water, potentially a cause of inter-regional conflicts and migration on an unimaginable scale, could prove to be one of the elements for peace and stable communities through mutual knowledge exchange and support. The concept of Science Diplomacy is a powerful one, uniting the politician, the diplomat, the scientist and the technician in a common cause, reducing inter-regional tensions and avoiding potential armed conflicts. Indeed, there is no one who cannot or should not be involved in this most noble of enterprises.

The relationship between science and policy is an old one, and often closely related to the world of culture, which in turn serves as a neutral element capable of reinforcing coalition. It is no coincidence that the Urban Water Atlas for Europe has been created not only by scientists and researchers, but also artists, politicians, municipal stakeholders and children who have provided the power of their imagination to produce many of the illustrations in this book. The atlas’ production proves that a new approach to integrating science, policy and engagement is possible.
